# Clinical Volume and Perioperative Outcomes of Hiatal Hernia Repair Within the Society of Thoracic Surgeons-General Thoracic Surgery Database

**DOI:** 10.1097/AS9.0000000000000622

**Published:** 2025-10-02

**Authors:** Jeremy J. Rosenbaum, Hailey A. Theeuwen, Shefali Varma, Eric S. Kawaguchi, Li Ding, Brooks V. Udelsman

**Affiliations:** From the * Keck School of Medicine of USC, Los Angeles, CA; † Department of Surgery, Keck School of Medicine of USC, Los Angeles, CA; ‡ Department of Population and Public Health Sciences, Keck School of Medicine of USC, Los Angeles, CA.

**Keywords:** hiatal hernia, invasive surgery, large dataset analysis, minimally quality improvement, national database, volume outcome relationship

## Abstract

**Background:**

This study investigates the relationship between clinical center operative volume and perioperative outcomes for elective hiatal hernia repairs.

**Methods:**

Patients receiving an elective hiatal hernia repair within the Society of Thoracic Surgeons-General Thoracic Surgery Database (2018–2023) were included. Patients with a cancer diagnosis or achalasia were excluded. Participant centers were categorized into low-, medium-, and high-volume tertiles based on annual elective hiatal hernia operative volume. Primary outcomes were 30-day morbidity and reoperation. Secondary outcomes included mortality and 30-day readmission. Multivariable regressions were performed to adjust for covariates, including sociodemographics, comorbidities, and hernia characteristics.

**Results:**

Among 174 centers, 13,658 elective hiatal hernia repairs were performed. A total of 295 (2.2%), 1714 (12.5%), and 11,649 (85.3%) repairs were performed at low-, medium-, and high-volume centers, respectively. Mortality within 30 days was <0.5% and did not differ by center volume. There was a stepwise decrease in 30-day morbidity (22.4% vs 18.4% vs 14.0%; *P* < 0.001), reoperation (4.7% vs. 2.7% vs 1.7%; *P* <0.001), and readmission (7.8% vs 7.3% vs. 5.8%; *P* < 0.001) when comparing low-, medium-, and high-volume centers. Minimally invasive approaches were more common at high-volume centers (94.4% vs 81.5% vs 82.1%; *P* < 0.001), and length of stay was shorter (2 days vs 3 days vs 3 days; *P* < 0.001). These differences remained significant for 30-day morbidity and 30-day reoperation in multivariable analysis.

**Conclusions:**

Perioperative outcomes after hiatal hernia repair were significantly improved when treatment occurred at high-volume centers. Referral to high-volume centers is encouraged for elective repairs.

## Graphical abstract

**Figure:**
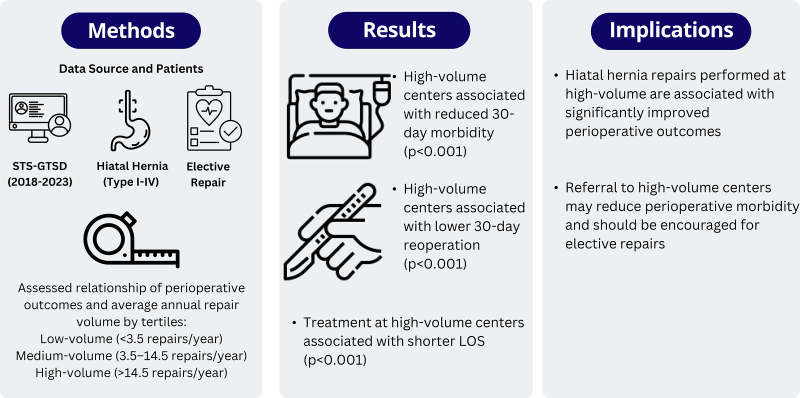

## BACKGROUND

Hiatal hernias range from small sliding types to giant ones involving multiple organs.^1,2^ Hiatal hernias are commonly observed in older individuals and those with conditions causing increased intra-abdominal pressure.^3,4^ The typical presentation leading to an evaluation is gastroesophageal reflux disease. Elective repair is recommended for symptomatic disease or prophylactically to prevent volvulus in type II hiatal hernias.^5^

Hiatal hernia repairs range in difficulty. Many studies have demonstrated a relationship between operative volume and improved outcomes for complex surgical operations such as pulmonary lobectomy, esophagectomy, and pancreaticoduodenectomy.^6,7^ However, for operations with nonmalignant indications and short postoperative length of stay (LOS), such as carotid endarterectomy, this relationship is less clear.^8,9^ Other factors that influence outcomes—including age and comorbidities—have already been well-established in the literature.^10,11^ Understanding these factors is essential for identifying modifiable targets to improve quality.

This study investigates the relationship between clinical center operative volume and perioperative outcomes for elective hiatal hernia repairs. We chose to utilize the Society of Thoracic Surgeons-General Thoracic Surgery Database (STS-GTSD), which is a robust dataset containing information on preoperative evaluation, intraoperative details, hernia characteristics, and specific outcomes related to hiatal hernia repair that were not available in prior studies on this topic. We aimed to determine whether patients treated at high-volume centers (top third by annual case volume) experience reduced 30-day morbidity and fewer reoperations compared with those treated at low- and moderate-volume centers.

## PATIENTS AND METHODS

### Data Source

The STS-GTSD is the most robust thoracic surgical database, with over 800,000 procedure records from 287 participating sites and more than 1000 participating surgeons.^12^ The STS-GTSD is a national clinical registry with detailed, clinician-abstracted data, which provides greater granularity than administrative databases. This allowed for more accurate identification of procedural details, patient risk factors, hernia characteristics, and perioperative outcomes. Use of the STS-GTSD for this project was approved by the Institutional Review Board and the STS-GTSD Database Access and Publications committees.

### Inclusion and Exclusion Criteria

The database was queried for patients undergoing elective hiatal hernia repair between 2018 and 2023. Inclusion criteria were age ≥18, diagnosed hiatal hernia, and operative repair. Exclusions included esophageal or lung cancer, achalasia, urgent/emergent repairs, an American Society of Anesthesiologists (ASA) score >4, and missing 30-day mortality data. We chose to exclude urgent/emergent repairs and patients with an ASA score >4 (moribund patients not expecting survival without an operation) to mitigate potentially confounding impacts of outlier patients.

### Hospital-Volume Stratification

Participant centers were defined as a single entity that signed a participation agreement with the STS and submitted a single data file. Certain participant centers may consist of data from multiple associated hospitals.^13^ Centers were divided into tertiles (low, moderate, and high) based on annual elective hiatal hernia repair volume, and patients were stratified by the annual operative volume of their surgical center. This method accounts for perturbations in volume due to the COVID-19 pandemic. Previous volume-outcome analyses have validated the use of center volume tertiles in other low-mortality procedures.^14^ By distributing centers into tertiles, we aimed to better capture a potential gradient in outcomes and reflect real-world variability in institutional volume. An exploratory receiver operating characteristic analysis was conducted as a supplemental evaluation, displaying a locally weighted scatterplot smoothing (LOESS) with logit transformation of the outcome. Key inflection points on the curves were identified to determine optimal volume thresholds associated with improved perioperative outcomes. Preliminary analyses revealed that a medium-volume cohort would encompass the 10 cases/year volume threshold, and a high-volume cohort would consist of centers performing an average annual case volume of ≥14.5, with a median of ~25 repairs per year.

### Covariates

Covariates included age, sex, race, ethnicity, body mass index (BMI), insurance status, Eastern Cooperative Oncology Group score (ECOG), ASA classification, and comorbidities. Hernia characteristics included prior repair history, symptoms, proton pump inhibitor (PPI) use and response (complete, moderate, no relief), esophagitis, and hernia type (I-IV). Captured operative characteristics included approach, fundoplication type, and use of LINX procedure, gastropexy, or mesh.

### Primary and Secondary Outcomes

Primary outcomes were 30-day morbidity and reoperation rates. Morbidity was defined within the STS-GTSD database.^13^ Secondary outcomes included 30-day mortality, readmission, endoscopic intervention, radiographic recurrence, symptom recurrence, LOS, operative duration (time entering operating room to time exiting), conversion from minimally invasive to open procedures, and disposition.

### Subgroup Analysis

A subgroup analysis was performed, excluding type I hiatal hernias to evaluate a relationship between more complex operative repairs and outcomes. Univariable and multivariable regressions were compared with our primary analysis to evaluate whether volume-outcome relationships persisted.

### Statistical Analysis

Patient, hernia, and operative characteristics were compared using Pearson χ^2^ and Wilcoxon rank-sum tests, as appropriate. Univariable and multivariable regressions were used to determine the association between outcomes and participant center operative volume. Linear regression was used for continuous outcomes (expressed as coefficients with 95% confidence intervals [CIs]). Linearity assumption was checked by residual plots. Logistic regression was used for binary outcomes ≤10% (expressed as odds ratios [OR] with 95% CIs). Hosmer–Lemeshow goodness-of-fit test was used for overall model fitting. For binary outcomes affecting >10% of the population, Poisson regression with robust variance was used to calculate risk ratios (RRs) with 95% CIs.^15^ Negative binomial regression was used for postsurgery LOS, treated as count, which account for overdispersion. Generalized estimation equation was incorporated in all models to adjust for hospital clustering. We also conducted subgroup analysis excluding type I hiatal hernia to test our results robustness. Same approach was used in the subgroup analysis as the main analysis. To adjust for potential confounders, multivariable analysis models included the following clinically relevant variables: center volume (tertiles), sociodemographics, comorbidities, and hernia characteristics. T-sided *P* values <0.05 were considered statistically significant. Data analyses were conducted using Stata version 18.0 (StataCorp LLC) and SAS version 9.4 (SAS Institute) for LOESS curves, in accordance with journal guidelines.

## RESULTS

**FIGURE 1.**
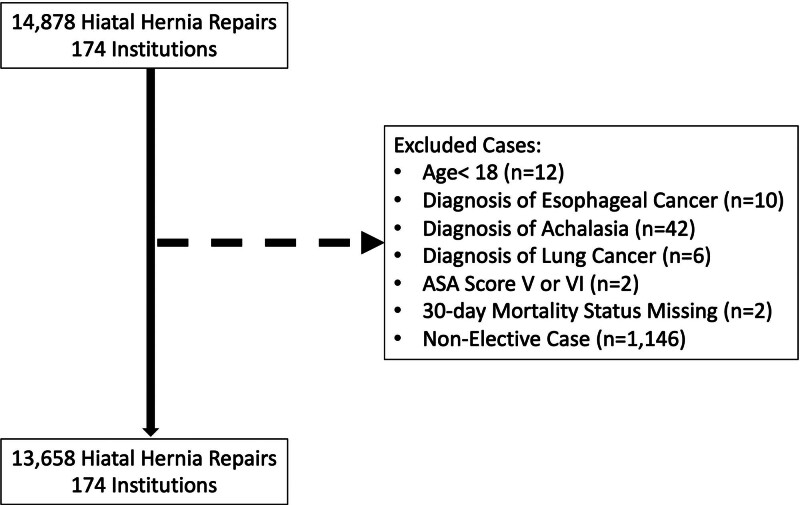
Inclusion and exclusion criteria.

Within the STS-GTSD, 14,625 patients received 14,878 procedures for hiatal hernia repairs at 174 centers between 2018 and 2023. After application of exclusion criteria, the final cohort consisted of 13,480 patients who received 13,658 procedures at 174 centers (Fig. 1).

### Volume of Hiatal Hernia Repairs Among Participant Centers

**FIGURE 2.**
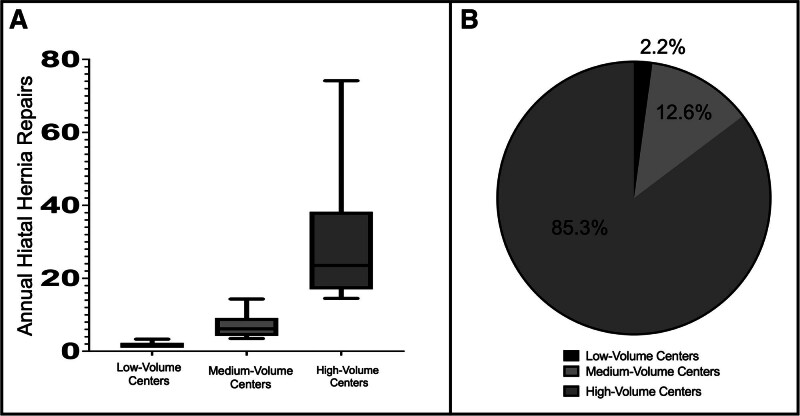
Annual number of hiatal hernia repairs (A) and percent of total hernia repairs (B) performed at low-, medium-, and high-volume centers. Box represents median and interquartile range; whiskers, 1.5 times the interquartile range. Four outliers in the high-volume group are not shown due to scale.

Participant centers were split into tertiles based on average annual hiatal hernia repair volume (Fig. 2). Average annual volume was calculated by dividing the number of hiatal hernias performed during the 6-year study period (2018–2023) by the number of years the participant center contributed data. Of the 174 centers, 60 were low-volume (<3.5 repairs/year), 56 were medium-volume (3.5–14.5 repairs/year), and 58 were high-volume (>14.5 repairs/year). Median annual repairs were 1.9, 6.2, and 25.7 for low-, medium-, and high-volume centers, respectively. Among participating centers, 21 (12.1%) performed only a single repair annually, and 78 (44.8%) performed fewer than 5 repairs per year.

### Patient Characteristics

The mean age was 64.1 years, the average BMI was 29.5, and 85.4% of patients had an ECOG score of 0–1 (Table 1). The majority of patients were female (73.0%). These variables did not differ significantly between participant center volume cohorts. Racial and ethnic distributions varied by less than 5% across center volumes, and insurance status differed by less than 3%.

**TABLE 1.** Patient Characteristics Compared Between Low-, Medium-, and High-Volume Centers

.	Low-Volume Centers (60 Centers, 295 Repairs)	Medium-Volume Centers (56 Centers, 1714 Repairs)	High-Volume Centers (58 Centers, 11,649 Repairs)	*P*
Age, mean (SD)	65.23 (11.4)	65.90 (12.3)	63.85 (13.2)	<0.001
Age categories				<0.001
<65	130 (44.1%)	678 (39.6%)	5257 (45.1%)	
65–80	138 (46.8%)	851 (49.6%)	5407 (46.4%)	
>80	27 (9.2%)	185 (10.8%)	985 (8.5%)	
Male	79 (26.8%)	467 (27.2%)	3147 (27.0%)	
Race				0.01
White	247 (83.7%)	1557 (90.8%)	10,269 (88.2%)	
Black	20 (6.8%)	71 (4.1%)	590 (5.1%)	
Asian	5 (1.7%)	9 (0.5%)	121 (1.0%)	
Other	18 (6.1%)	47 (2.7%)	446 (3.8%)	
Unknown	5 (1.7%)	30 (1.8%)	223 (1.9%)	
Hispanic	29 (9.8%)	85 (5.0%)	552 (4.7%)	<0.001
BMI categories				0.12
<30	171 (58.0%)	999 (58.3%)	6528 (56.0%)	
30–34.9	85 (28.8%)	479 (27.9%)	3492 (30.0%)	
≥35	38 (12.9%)	227 (13.2%)	1605 (13.8%)	
Unknown	1 (0.3%)	9 (0.5%)	24 (0.2%)	
Insurance status				<0.001
Medicare	154 (52.2%)	989 (57.7%)	6019 (51.7%)	
Medicaid	15 (5.1%)	94 (5.5%)	753 (6.5%)	
Private	111 (37.6%)	588 (34.3%)	4575 (39.3%)	
Military	7 (2.4%)	21 (1.2%)	175 (1.5%)	
Self-pay	7 (2.4%)	6 (0.4%)	57 (0.5%)	
Unknown	1 (0.3%)	16 (0.9%)	70 (0.6%)	
ECOG category				<0.001
0	178 (60.3%)	918 (53.6%)	7109 (61.0%)	
1	59 (20.0%)	455 (26.5%)	2956 (25.4%)	
≥2	12 (4.1%)	53 (3.1%)	460 (3.9%)	
Unknown	46 (15.6%)	288 (16.8%)	1124 (9.6%)	
ASA				0.12
I	1 (0.3%)	5 (0.3%)	54 (0.5%)	
II	98 (33.2%)	540 (31.5%)	4016 (34.5%)	
III	196 (66.4%)	1169 (68.2%)	7579 (65.1%)	
Comorbidities				
Cardiopulmonary disease	173 (58.6%)	1088 (63.5%)	7232 (62.1%)	0.24
Peripheral arterial disease	46 (15.6%)	305 (17.8%)	1825 (15.7%)	0.08
Diabetes	44 (14.9%)	184 (10.7%)	1391 (11.9%)	0.09
Dialysis	0 (0.0%)	2 (0.1%)	7 (0.1%)	0.63
Liver dysfunction	3 (1.0%)	33 (1.9%)	147 (1.3%)	0.07
Active smoker	17 (5.8%)	98 (5.7%)	686 (5.9%)	0.96
Substance abuse history	12 (4.1%)	68 (4.0%)	556 (4.8%)	0.3
Major psychiatric disorder	67 (22.7%)	573 (33.4%)	4048 (34.7%)	<0.001

The most common comorbidities across all center cohorts were cardiopulmonary diseases and major psychiatric disorders. High-volume centers, compared with medium- and low-volume centers, saw more patients with major psychiatric disorders (34.7% vs 33.4% vs 22.7%; *P* < 0.001). Other comorbidities did not differ significantly by center volume.

### Hernia and Operative Characteristics

Hernia and operative characteristics are listed in Table 2. Redo hiatal hernia operations were performed in 14.1% of the repairs, with less than 5% variation between center volumes. The most common symptoms were heartburn (74.0%), regurgitation (66.5%), and dysphagia (60.4%). Patients at high- and medium-volume centers were less likely to be asymptomatic compared with those at low-volume centers (1.4% vs 1.6% vs 3.1%; *P* = 0.01).

**TABLE 2.** Hernia and Operative Characteristics

	Low-Volume Centers (60 Centers, 295 Repairs)	Medium-Volume Centers (56 Centers, 1714 Repairs)	High-Volume Centers (58 Centers, 11,649 Repairs)	*P*
Redo operation	36 (12.2%)	299 (16.5%)	1594 (13.6%)	0.01
Symptoms				
Asymptomatic	9 (3.1%)	28 (1.6%)	167 (1.4%)	0.07
Heartburn	168 (70.0%)	868 (69.2%)	6988 (74.7%)	<0.001
Cough	76 (38.2%)	296 (30.3%)	2512 (33.1%)	0.06
Regurgitation	137 (61.2%)	833 (66.9%)	6089 (66.6%)	0.23
Hoarseness	30 (16.4%)	104 (11.9%)	774 (11.1%)	0.08
Dysphagia	107 (52.5%)	761 (63.6%)	5400 (60.1%)	0.01
Sore throat	12 (6.9%)	70 (8.1%)	448 (6.5%)	0.21
Epigastric/chest pain	115 (53.0%)	815 (66.6%)	5375 (60.1%)	<0.001
Asthma	27 (14.8%)	120 (13.5%)	1295 (18.2%)	<0.01
Early satiety	67 (34.7%)	381 (36.5%)	2152 (28.1%)	<0.001
Reflux laryngitis	20 (10.9%)	45 (5.3%)	589 (8.4%)	<0.01
Anemia	32 (17.8%)	217 (22.8%)	1921 (25.8%)	0.01
PPI use	210 (71.2%)	1292 (75.4%)	9485 (81.4%)	<0.001
PPI response				0.18
Complete relief	24 (11.8%)	138 (10.9%)	1235 (13.4%)	
Partial response	114 (55.9%)	723 (57.3%)	5157 (55.8%)	
No response	66 (32.4%)	401 (31.8%)	2843 (30.8%)	
Esophagitis	57 (19.3%)	294 (17.2%)	2316 (19.9%)	0.03
Barrett’s esophagus	9 (3.6%)	61 (4.5%)	296 (3.0%)	0.01
Hernia type				<0.001
I	66 (22.4%)	302 (17.6%)	3048 (26.2%)	
II	63 (21.4%)	312 (18.2%)	1505 (12.9%)	
III	107 (36.3%)	772 (45.0%)	5892 (50.6%)	
IV	59 (20.0%)	328 (19.1%)	1204 (10.3%)	
Manometry performed	106 (35.9%)	509 (29.8%)	4475 (38.5%)	<0.001
Motility				<0.001
Normal	68 (66.7%)	349 (70.8%)	3076 (69.7%)	
Decreased	30 (29.4%)	126 (25.6%)	1230 (27.9%)	
Aperistalsis	4 (3.9%)	18 (3.7%)	105 (2.4%)	
Operative approach				<0.001
Laparoscopic	86 (29.2%)	543 (31.7%)	6322 (54.3%)	
Robotic-assisted	156 (52.9%)	854 (49.8%)	4677 (40.1%)	
Open	53 (18.0%)	317 (18.5%)	650 (5.6%)	
Fundoplication				0.01
None	68 (23.1%)	473 (27.6%)	3103 (26.6%)	0.01
Partial	129 (56.8%)	763 (61.5%)	5166 (60.4%)	
Complete	95 (41.9%)	475 (38.3%)	3363 (39.4%)	
LINX procedure	1 (0.8%)	16 (1.8%)	192 (3.9%)	<0.01
Gastroplasty	34 (11.6%)	268 (15.7%)	1344 (11.5%)	<0.001
Mesh reinforcement	45 (15.3%)	255 (14.9%)	2751 (23.6%)	<0.001
Relaxing diaphragmatic incision	15 (5.1%)	58 (3.4%)	369 (3.2%)	0.17

PPI usage decreased stepwise from 81.4% to 75.4% to 71.2% for high-, medium-, and low-volume centers (*P* < 0.001). High-volume centers had a higher proportion of type I (26.2% vs 17.6% vs 22.4%; *P* < 0.001) and type III hiatal hernias (50.6% vs 45.0% vs 36.3%; *P* < 0.001), but fewer type II (12.9% vs 18.2% vs 21.4%; *P* < 0.001) and type IV hiatal hernias (10.3% vs 19.1% vs 20.0%; *P* < 0.001) (Table 2).

At high-volume centers, minimally invasive approaches were significantly more common (94.4% vs 81.5% vs 82.1%; *P* < 0.001), and LINX procedures (3.9% vs 1.8% vs 0.8%; *P* < 0.01) and mesh reinforcement (23.6% vs 14.9% vs 15.3%; *P* < 0.001) were more frequently used.

### Primary and Secondary Outcomes

Treatment at high-volume centers was associated with reduced 30-day morbidity (14.0% vs 18.4% vs 22.5%; *P* < 0.001) and lower reoperation rates (1.7% vs 2.7% vs 4.7%; *P* < 0.001) (Table 3). Multivariable Poisson regression showed significantly greater rates of 30-day morbidity among low-volume centers (RR 0.31; 95% CI: 0.04–0.58; *P* = 0.03) and medium-volume centers (RR 0.23; 95% CI: 0.05–0.43; *P* = 0.02). In multivariable logistic regression, treatment at high-volume centers remained associated with reduced reoperation (Table 4). The most common complications were atrial arrhythmias, pleural effusions, urinary tract infections, delirium, surgical site infections, and pneumonias (Supplementary Table 1, see https://links.lww.com/AOSO/A544).

**TABLE 3.** Univariable Analysis for Primary and Secondary Outcomes

	Low-Volume Centers (60 Centers, 295 Repairs)	Medium-Volume Centers (56 Centers, 1714 Repairs)	High-Volume Centers (58 Centers, 11,649 Repairs)	*P*
	Primary outcomes			
30-day morbidity	66 (22.4%)	315 (18.4%)	1635 (14.0%)	<0.001
30-day reoperation	14 (4.7%)	47 (2.7%)	200 (1.7%)	<0.001
	Secondary outcomes			
30-day mortality	1 (0.3%)	7 (0.4%)	49 (0.4%)	0.98
Conversion from MI to open procedure	9 (3.1%)	29 (1.7%)	73 (0.6%)	<0.001
Operative duration in minutes, median (IQR)	252.5 (203, 308)	256 (195, 327)	205 (161, 267)	<0.001
LOS, median (IQR)	3 (2, 5)	3 (2, 4)	2 (1, 4)	<0.001
30-day readmission	23 (7.8%)	125 (7.3%)	676 (5.8%)	<0.001
30-day radiographic recurrence	6 (2.4%)	66 (4.2%)	134 (1.3%)	<0.001
Discharge home	279 (94.6%)	1618 (94.4%)	11365 (97.6%)	<0.001

MI, minimally invasive; IQR, interquartile range.

**TABLE 4.** Multivariable Regressions for Primary and Secondary Outcomes

Outcome	Low- Versus High-Volume Tertiles	95% CI	*P*	Medium- Versus High-Volume Tertiles	95% CI	*P*
30-day morbidity (OR)*	1.513	[1.056, 2.167]	0.024	1.357	[1.052, 1.750]	0.019
30-day reoperation (OR)*	4.649	[1.975, 10.94]	0.000	2.183	[1.241, 3.839]	0.007
30-day readmission (OR)*	0.874	[0.479, 1.593]	0.660	1.100	[0.736, 1.643]	0.642
30-day radiographic recurrence (OR)*	2.355	[0.865, 6.411]	0.094	3.033	[1.353, 6.800]	0.007
30-day symptom recurrence (OR)*	1.550	[0.846, 2.839]	0.156	1.015	[0.625, 1.651]	0.951
Discharge to home (RR)†	0.967	[0.931, 1.006]	0.095	0.980	[0.959, 1.002]	0.069
30-day mortality (OR)*	1.791	[0.205, 15.67]	0.598	1.514	[0.375, 6.108]	0.560
Conversion from MI to open procedure (OR)*	4.172	[1.349, 12.90]	0.013	2.464	[1.147, 5.295]	0.021
Length of stay (IRR)‡	1.332	[1.042, 1.701]	0.022	1.500	[1.204, 1.869]	<0.001
Operative duration (coefficient)§	39.13	[12.56, 65.69]	0.004	36.37	[10.94, 61.81]	0.005

95% CI in brackets.

* OR for logistic regression.

† RR for Poisson models with robust variance.

‡ Incidence Rate Ratios (IRR) for negative binomial models.

§ Mean differences in minutes (raw coefficient) for linear regression.

High-volume centers had fewer conversions to open procedures (0.6% vs 1.7% vs 3.1%; *P* < 0.001) and shorter operative durations (205 vs 256 vs 252.5 minutes; *P* < 0.001). The 30-day mortality rate was <0.5% and did not differ by volume. Univariable analysis showed that high-volume centers had shorter LOS, reduced 30-day readmission rates, fewer 30-day radiographic recurrences, and higher rates of discharge home. These trends persisted in multivariable analysis but did not reach significance for LOS, readmission, and discharge to home.

### Participant Center-Volume Analysis

**FIGURE 3:**
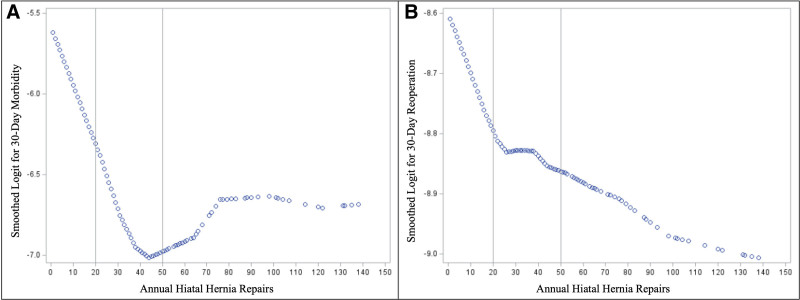
Locally weighted scatterplot smoothing with logit transformation examining volume thresholds associated with decreased 30-day morbidity (A) and 30-day mortality (B).

To determine a volume threshold, exploratory analysis was performed. Key inflection points were identified LOESS plots for the primary outcomes of 30-day morbidity and 30-day reoperation (Fig. 3). The nadir for 30-day morbidity occurred at ~40 repairs per year, while the nadir for 30-day reoperation continued to decrease as annual volume increased; however, there were inflection points in the curve at ~20 cases per year and ~40 cases per year. Among high-volume centers, 39 (67%) met a threshold of 20 cases per year, and 15 (25.8%) met a threshold of 40 cases per year.

### Subgroup Analysis

Given the overrepresentation of type I hiatal hernias among high-volume centers, a subgroup analysis limited to type II, III, and IV hiatal hernias was performed to evaluate whether volume-outcome relationships persisted. Univariable and multivariable analyses closely reflected our initial analyses, with high-volume centers demonstrating reduced morbidity, 30-day reoperation, LOS, and operative duration. Of note, the volume-outcome relationship became marginally more pronounced among our primary outcomes on multivariable analysis (Supplementary Table 2, see https://links.lww.com/AOSO/A544).

## DISCUSSION

In this study of 13,658 hiatal hernia repairs at 174 STS-GTSD participant centers, we found that high-volume centers had favorable outcomes across a wide range of metrics, including 30-day morbidity, reoperation, and readmission rates. Patients at high-volume centers also had shorter hospital stays, higher rates of minimally invasive repairs, and increased rates of being discharged home. In this study, we explored annual volume through tertiles divided into low-, medium-, and high-volume centers based on annual volume. This empiric classification was further supported by exploratory participant center-volume analysis, which confirmed thresholds that correlated with the high-volume tertile.

Many surgical procedures yield better outcomes when performed at high-volume centers.^6,7^ Cardiothoracic operations with established volume threshold relationships include esophagectomy, pulmonary lobectomy, open aortic procedures, and mitral valve repair/replacement.^16^ Specific to hiatal hernias, Whealon et al^17^ found a small but significant relationship between volume and decreased mortality for laparoscopic repairs. This study builds on that work by including a broader spectrum of elective hiatal hernia repairs, demonstrating significant reductions in morbidity and early reoperation rates.

We were unable to replicate the mortality difference seen by Whealon et al. Several explanations may explain this discrepancy, including differences in study time period, inclusion criteria, and volume thresholds. Additionally, we used the STS-GTSD, which is specific to thoracic surgeons, whereas their study used the national readmissions dataset, which includes all surgeons. Mortality across our cohort was rare, occurring in <0.5% of cases across all volume tertiles. While many outcomes-based initiatives focus on inpatient mortality rates, surgical advancements have made mortality a less reliable measurement of quality.^18^ For procedures with low-mortality but high-complication rates, morbidity and readmission rates may offer more meaningful insights into quality.^19^

The volume tertiles in this study were based on prespecified study design criteria, akin to prior work using cutoffs for pulmonary lobectomy outcomes.^14^ We also performed an exploratory analysis for the 2 prior outcomes looking at various volume cutoffs, finding a nearly linear decrease in morbidity with volume up to a nadir of 40 cases annually. Other studies evaluating volume-outcome relationships in the context of thoracic surgery have identified favorable outcomes among patients treated at centers performing 40 or more annual cases.^20^ For reoperation, the curve was more complex but still inversely correlated with volume. While our high-volume cutoff was 14.5 cases annually, the median for high-volume centers was 25.7, approximating the exploratory analysis.

Minimum-volume standards have been explored in other areas of surgery.^21^ While these thresholds can improve quality, they may also increase travel burden and costs.^22^ Vulnerable populations, including racial minorities, the elderly, and those with limited insurance may be most affected.^23^ Although this study supports the use of volume thresholds, it cannot detail how this might impact patient access to surgery. Whether the increased morbidity at low-volume centers justifies the potential access issues for patients who cannot travel to high-volume centers is unknown.

We did note some heterogeneity in the repairs, with 26.7% of patients not receiving a fundoplication. Among those who did, two-thirds received a partial wrap and one-third a complete wrap. Multivariable analysis showed no significant differences in primary outcomes based on the type of repair (Supplementary Tables 3–4, see https://links.lww.com/AOSO/A544). Similarly, the use of reinforcing mesh or gastroplasty did not correlate with perioperative outcomes. Other studies have explored the long-term effects of both of these maneuvers on morbidity and recurrence.^24,25^

Strengths of this work include the use of the STS-GTSD, a well-established database with independent auditing for quality assurance. Using detailed clinician-abstracted data, this national clinical registry provides greater granularity than administrative databases used in prior studies. This allowed for more accurate identification of procedural details, patient risk factors, hernia characteristics, and perioperative outcomes. Furthermore, our primary endpoints–30-day morbidity and reoperation–are accurately captured in the STS-GTSD. This contrasts with prior NIS-based studies where definitions may be less reliable due to coding variability. These results have important implications. While mortality may be minimally affected by treatment at low-volume centers, morbidity is significantly affected and nearly doubles between low- and high-volume centers. Referral of elective hiatal hernia repairs may substantially reduce morbidity, result in higher quality care, and potential savings for the health care system. Patients, clinicians, hospitals, and insurers should be aware of this information and inform treatment decisions and policy accordingly.

A limitation of the STS-GTSD is that the data reflect only the practice of thoracic surgeons. National estimates suggest that the majority of hiatal hernia repairs, approximately 72% in a Vizient database analysis by Gambhir et al,^26^ are performed by general surgeons, often within minimally invasive or benign foregut practices that are not captured within the STS registry. As a result, our definition of high-volume centers reflects case volume within the STS cohort likely represents a subset of all hiatal hernia repairs performed nationally. It is possible that at low-volume STS centers, general surgeons perform many hiatal hernia repairs, but this data is uncaptured. However, if that were the case, we would not expect such a strong relationship between center volume and perioperative outcomes within the STS dataset. Furthermore, general and thoracic surgery services often operate in silos, with limited shared patient care, which may negate this type of institutional experience. These factors should be considered when interpreting the generalizability of our findings, and future studies using multispecialty datasets could clarify whether the observed volume-outcome association persists across a broader population of patients undergoing hiatal hernia repairs.

This study focused on elective hiatal hernia repairs for several reasons. First, determining the acuity and degree of illness in urgent or emergent cases is difficult and may introduce significant bias. Second, low-volume centers may be the best option for high-acuity patients. Patients who can transfer to high-volume centers are likely less ill, potentially skewing outcomes. In contrast, elective cases allow for optimal center selection.

The STS-GTSD contains robust data on 30-day outcomes but limited data on long-term outcomes, which are crucial when evaluating the efficacy of hiatal hernia repair. However, we expect the differences observed in 30-day quality, especially those related to morbidity and reoperation, to persist or even become more pronounced over time. We also cannot account for differences in postoperative care, such as diet advancement, which may contribute to LOS. While center volume is a key factor in quality, it is not the only one, and we acknowledge that some low-volume centers may have excellent outcomes, just as some high-volume centers may have relatively poor outcomes.^27^ Finally, we used the ECOG score as a functional status measure, recognizing that although it was designed for patients with malignancy, it can also serve as an estimate of functional status in this cohort.

To conclude, our study illustrates that hiatal hernia repairs performed at high-volume centers by thoracic surgeons are associated with significantly improved perioperative outcomes. Despite a low-mortality rate (<0.5%), hiatal hernia repair has a relatively high morbidity rate (14.8%). Referral to high-volume centers may reduce perioperative morbidity and should be encouraged for elective repairs.

## Acknowledgments

The data for this research were provided by The Society of Thoracic Surgeons’ National Database Participant User File Research Program. Data analysis was performed at the investigators’ institution(s). The views and opinions presented in this article are solely those of the author(s) and do not necessarily represent those of The Society of Thoracic Surgeons. J.J.R.: Conceptualization, Methodology, Software, Validation, Formal Analysis, Investigation, Data Curation, Writing – Original Draft, Writing – Review & Editing, Visualization. H.A.T.: Writing – Review & Editing, Visualization. S.V.: Writing – Review & Editing, Visualization. E.S.K.: Software, Validation, Formal Analysis, Resources, Data Curation, Writing – Review & Editing. L.D.: Software, Validation, Formal Analysis, Resources, Data Curation, Writing – Review & Editing. B.V.U.: Conceptualization, Methodology, Software, Validation, Formal Analysis, Investigation, Resources, Data Curation, Writing – Review & Editing, Visualization, Supervision, Project Administration, Funding Acquisition.

## Supplementary Material


