# Supplementary material for: Clinical Volume and Perioperative Outcomes of Hiatal Hernia Repair Within the Society of Thoracic Surgeons-General Thoracic Surgery Database
Source: Ann Surg Open. 2025 Oct 2;6(4):e622. doi: 10.1097/AS9.0000000000000622 (PMC12727398; doi:10.1097/AS9.0000000000000622)
Supplement: Supplementary file 1 [file as9-6-e622-s001.pdf]

Supplementary Table 1: Post-operative morbidities

	Low-Volume Centers (60 centers, 295 repairs)	Medium-Volume Centers (56 centers, 1714 repairs)	High-Volume Centers (58 centers, 11,649 repairs)	p-value
Pneumonia	4 (1.4%)	26 (1.5%)	81 (0.7%)	<0.01
Empyema	3 (1.0%)	12 (0.7%)	14 (0.1%)	<0.001
Effusion requiring drainage	8 (2.7%)	41 (2.4%)	182 (1.6%)	0.02
Post-operative bronchoscopy	1 (0.3%)	10 (0.6%)	16 (0.1%)	<0.001
Pneumothorax requiring post-operative chest tube	8 (2.7%)	18 (1.1%)	98 (0.8%)	<0.01
Acute respiratory distress syndrome	1 (0.3%)	4 (0.2%)	13 (0.1%)	0.26
Respiratory failure requiring mechanical ventilation	5 (1.7%)	19 (1.1%)	105 (0.9%)	0.29
Deep vein thrombosis/Pulmonary embolism	3 (1.0%)	26 (1.5%)	112 (1.0%)	0.10
Post-operative transfusion	9 (3.1%)	31 (1.8%)	119 (1.0%)	<0.001
Myocardial infarction	0 (0.0%)	0 (0.0%)	20 (0.2%)	0.18
Air leak >5 post-operative days	2 (0.7%)	3 (0.2%)	5 (<1%)	<0.001
Atrial arrythmia requiring intervention	9 (3.1%)	56 (3.3%)	230 (2.0%)	<0.01
Ventricular arrythmia requiring intervention	0 (0.0%)	8 (0.5%)	7 (0.1%)	<0.001
Ileus	2 (0.7%)	21 (1.2%)	80 (0.7%)	0.06
Urinary track infection	5 (1.7%)	21 (1.2%)	121 (1.0%)	0.46
Surgical site infection	6 (2.0%)	17 (1.0%)	107 (0.9%)	0.15
Sepsis	4 (1.4%)	12 (0.7%)	36 (0.3%)	<0.01
Other infection	5 (1.7%)	27 (1.6%)	70 (0.6%)	<0.001
Stroke	4 (1.4%)	3 (0.2%)	19 (0.2%)	<0.001
Delirium	3 (1.0%)	27 (1.6%)	103 (0.9%)	0.03
Acute renal failure	3 (1.0%)	8 (0.5%)	14 (0.1%)	<0.001
Chest tube on discharge from hospital	2 (0.7%)	20 (1.2%)	32 (0.3%)	<0.001
Discharge with new home oxygen requirement	6 (2.0%)	73 (4.3%)	424 (3.6%)	0.14

**Supplementary Table 2: Multivariable regressions for primary and secondary outcomes: subgroup analysis excluding type I hiatal hernias**						
**Outcome**	**Low vs High Volume Tertiles**	**95% CI**	**p-value**	**Medium vs High Volume Tertiles**	**95% CI**	**p-value**
30-Day Morbidity (OR) †	**1.492 ***	**[1.028, 2.165]**	**0.035**	**1.351**	**[1.031, 1.772]**	**0.029**
30-Day Reoperation (OR) †	**5.259 *****	**[2.044, 13.53]**	**0.001**	**2.563**	**[1.433, 4.584]**	**0.002**
30-Day Readmission (OR) †	**0.812**	**[0.418, 1.579]**	**0.540**	**1.028**	**[0.673, 1.571]**	**0.898**
30-Day Radiographic Recurrence (OR) †	**1.717**	**[0.567, 5.198]**	**0.339**	**3.234**	**[1.473, 7.099]**	**0.003**
30-Day Symptom Recurrence (OR) †	**1.855**	**[0.944, 3.645]**	**0.073**	**1.231**	**[0.757, 2.003]**	**0.402**
Discharge to Home (RR) ††	**0.952**	**[0.905, 1.003]**	**0.063**	**0.977**	**[0.953, 1.002]**	**0.067**
30-Day Mortality (OR) †	**2.366**	**[0.268, 20.87]**	**0.438**	**1.825**	**[0.392, 8.486]**	**0.443**
Conversion from MI to Open Procedure (OR) †	**2.611**	**[0.777, 8.772]**	**0.121**	**1.923**	**[0.917, 4.031]**	**0.083**
LOS (IRR) †††	**1.300**	**[1.005, 1.541]**	**0.034**	**1.331**	**[1.072, 1.425]**	**0.001**
Operative Duration (Coefficient) ††††	40.08	[10.79, 69.38]	0.007	40.09	[14.16, 66.02]	0.002

95% confidence intervals in brackets
† Odds Ratios (OR) for logistic regression
†† Risk Ratios (RR) for Poisson models with robust variance
††† Incidence Rate Ratios (IRR) for negative binomial models
†††† Mean differences in minutes (raw coefficient) for linear regression

Supplementary Table 3: Poisson regression for 30-day Morbidity

		Coefficient	95%-CI	p-value
Clinical Volume				
	Low-Volume	0.31	0.04 -- 0.58	0.03
	Medium-Volume	0.24	0.04 -- 0.43	0.02
	High-Volume	Ref.		
Age Category				
	<65			
	65-80	-0.03	-0.17 -- 0.11	0.66
	>80	0.38	0.21 -- 0.55	<0.001
Male		0.15		0.21
Race				
	White	Ref.		
	Black	0.28	-0.24 -- 0.79	0.30
	Asian	0.43	-0.11 -- 0.97	0.12
	Other	-0.45	-1.47 -- 0.56	0.38
	Unknown	0.13	-0.47 -- 0.72	0.67
Hispanic		-0.01	-0.26 -- 0.24	0.92
Insurance Status				
	Private	Ref.		
	Medicare	-0.13	-0.35 -- 0.10	0.28
	Medicaid	-0.11	-0.26 -- 0.04	0.16
	Military	-0.35	-0.91 -- 0.21	0.22
	Self-Pay	-0.50	-1.53 -- 0.52	0.34
	Unknown	-0.15	-0.76 -- 0.45	0.62
ECOG Score				
	0	Ref.		
	1	0.11	-0.06 -- 0.29	0.21
	≥2	0.52	0.34 -- 0.70	0.00
Unknown		0.38	0.10 -- 0.66	0.01
ASA Category				
	I	Ref.		
	II	0.57	-0.52 -- 1.66	0.31
	III	0.69	-0.38 -- 1.77	0.20
BMI Category				
	<30	Ref.		
	30-35	-0.05	-0.20 -- 0.09	0.48
	>35	-0.10	-0.38 -- 0.19	0.50
	Unknown	0.05	-0.64 -- 0.74	0.88
Comorbidities				

CHF	0.14	0.03 -- 0.25	0.02
PVD	0.08	-0.07 -- 0.23	0.27
DM	0.05	-0.09 -- 0.19	0.47
Liver Dysfunction	0.20	-0.19 -- 0.59	0.31
Major Psychiatric Disorder	0.22	0.10 -- 0.34	0.00
Active Smoker	-0.06	-0.26 -- 0.14	0.56
Polysubstance Abuse	0.25	0.09 -- 0.42	0.00
Hernia Type			
I	Ref.		
II	0.13	-0.12 -- 0.38	0.31
III	0.14	-0.06 -- 0.34	0.16
IV	0.53	0.27 -- 0.79	0.00
Hernia Symptoms			
Asymptomatic	-0.10	-0.48 -- 0.27	0.59
Heartburn	-0.11	-0.22-- -0.01	0.04
Cough	0.04	-0.05 -- 0.14	0.37
Hoarse	-0.08	-0.25 -- 0.08	0.33
Dysphagia	0.13	0.01 -- 0.24	0.04
Epigastric Pain	-0.02	-0.14 -- 0.09	0.71
Asthma	0.11	-0.10 -- 0.32	0.30
Early Satiety	0.05	-0.06 -- 0.16	0.40
Reflux Laryngitis	-0.25	-0.51 -- 0.01	0.06
Anemia	0.00	-0.12 -- 0.13	0.99
PPI Use	-0.04	-0.17 -- 0.08	0.49
Esophagitis	0.10	-0.06 -- 0.25	0.24
Barrett's Esophagus	0.01	-0.13 -- 0.16	0.84
Prior Hiatal Hernia Repair	0.35	0.16 -- 0.54	0.00
Fundoplication type			
None	Ref.		
Partial	-0.11	-0.30 -- 0.07	0.24
Complete	-0.05	-0.35 -- 0.24	0.73
Gastroplasty	-0.21	-0.46 -- 0.03	0.08
Mesh Reinforcement	-0.06	-0.22 -- 0.09	0.45

CI, confidence interval; ECOG, eastern cooperative oncology group; BMI, body mass index; CHF, congestive heart failure; PVD, peripheral vascular disease; DM diabetes; PPI, proton pump inhibitor

Supplementary Table 4: Logistic regression for reoperation within 30 days of index procedure

		Odds ratio	95%-CI	p-value
Clinical Volume				
	Low-Volume	4.13	1.92 -- 8.90	0.00
	Medium-Volume	2.47	1.48 -- 4.12	0.00
	High-Volume	Ref.		
Age Category				
	<65	Ref.		
	65-80	0.68	0.36 -- 1.31	0.25
	>80	0.59	0.21 -- 1.64	0.31
Male		0.68	0.43 -- 1.23	0.73
Race				
	White			
	Black	0.91	0.35 -- 2.37	0.85
	Asian	0.48	0.09 -- 2.41	0.37
	Other	3.09	0.52 -- 18.4	0.21
	Unknown	0.74	0.13 -- 4.11	0.73
Hispanic		2.01	0.35 -- 11.5	0.43
Insurance Status				
Private		Ref.		
	Medicare	0.48	0.14 -- 1.57	0.23
	Medicaid	0.91	0.48 -- 1.72	0.77
	Military	1.10	0.24 -- 2.21	0.89
	Self-Pay	1.90	0.22 -- 16.6	0.56
	Unknown	1.23	0.17 -- 9.01	0.84
ECOG Score				
	0	Ref.		
	1	1.48	0.85 -- 2.56	0.16
	≥2	0.58	0.14 -- 2.47	0.47
Unknown		1.75	0.75 -- 4.08	0.19
ASA Category				
	I	Ref.		
	II	1.74	1.31 -- 3.31	0.00
	III	1.55	1.17 -- 2.79	0.00
BMI Category				
	<30	Ref.		
	30-35	1.02	0.63 -- 1.65	0.93
	>35	0.42	0.19 -- 0.91	0.03
	Unknown	0.90	0.11 -- 5.42	

Comorbidities			
CHF	0.91	0.56 -- 1.46	0.69
PVD	0.97	0.56 -- 1.72	0.93
DM	1.52	0.78 -- 2.94	0.22
Liver Dysfunction	1.15	0.14 -- 9.50	0.90
Major Psychiatric Disorder	1.52	0.97 -- 2.36	0.07
Active Smoker	0.45	0.10 -- 2.06	0.31
Polysubstance Abuse	1.11	0.46 -- 2.69	0.81
Hernia Type			
I	Ref.		
II	1.06	0.48 -- 2.34	0.88
III	1.41	0.73 -- 2.69	0.31
IV	3.16	1.51 -- 6.64	0.00
Hernia Symptoms			
Asymptomatic	0.88	0.22 -- 3.49	0.86
Heartburn	1.00	0.64 -- 1.55	0.99
Cough	1.03	0.58 -- 1.80	0.91
Hoarse	0.47	0.19 -- 1.16	0.10
Dysphagia	0.91	0.55 -- 1.48	0.69
Epigastric Pain	0.73	0.46 --1.18	0.20
Asthma	0.91	0.48 -- 1.68	0.76
Early Satiety	0.82	0.46 -- 1.45	0.50
Reflux Laryngitis	1.07	0.33 -- 3.37	0.91
Anemia	1.59	0.87 -- 2.89	0.13
PPI Use	0.98	0.64 -- 1.49	0.94
Esophagitis	0.72	0.40 -- 1.31	0.28
Redo Operation	2.26	0.92 -- 5.54	0.08
Fundoplication type			
None	Ref.		
Partial	1.02	0.58 -- 1.78	0.94
Complete	1.18	0.68 -- 2.02	0.55
Gastroplasty	1.08	0.45 -- 2.58	0.86
Mesh Reinforcement	1.24	0.75 -- 2.03	0.40

ECOG, eastern cooperative oncology group; BMI, body mass index; CHF, congestive heart failure; PVD, peripheral vascular disease; DM diabetes; PPI, proton pump inhibitor
